# Probiotics and Diet in Rosacea: Current Evidence and Future Perspectives

**DOI:** 10.3390/biom15030411

**Published:** 2025-03-13

**Authors:** Marco Manfredini, Michele Barbieri, Margherita Milandri, Caterina Longo

**Affiliations:** 1 Department of Dermatology, University of Modena and Reggio Emilia, 41124 Modena, Italy; mbarbieri1996@gmail.com (M.B.); margheritamilandri@gmail.com (M.M.); caterina.longo@unimore.it (C.L.); 2 Azienda Unità Sanitaria Locale—IRCCS di Reggio Emilia, Skin Cancer Center, Policlinico di Modena, 41124 Modena, Italy

**Keywords:** rosacea, probiotics, foods, diet, retinoids

## Abstract

Rosacea is a common inflammatory skin disease, characterized by erythema, papules and pustules. The pathophysiology of rosacea remains unclear, but the complex interplay between environmental and genetic factors may act as a trigger to an abnormal innate immune response associated with a multifaceted neurovascular reaction. Increasing evidence suggests that the gut microbiota is significantly involved in the pathogenesis of rosacea, playing an important role in the inflammatory cutaneous response. Dysbiosis, small intestinal bacterial overgrowth, Helicobacter pylori infection and innate immune system dysregulation mutually contribute to the pathophysiology of rosacea, but more extensive future research is needed to better clarify their precise mechanisms of action. Many dietary triggers have been postulated for this disease; however, there is a lack of well-made and controlled studies able to undoubtedly demonstrate a causal relationship between rosacea and diet. We analyzed the available studies on the role of diet and gut microbiome in rosacea and the positive clinical effects reported by the current literature on probiotics, prebiotics, postbiotics and nutrients. Ultimately, this article improves our understanding of the gut–skin axis in rosacea, focusing on how probiotic supplementation and diet could improve the clinical management of patients affected by this common and debilitating disease.

## 1. Introduction

### 1.1. Rosacea Clinical Features and Types

Rosacea is a chronic, inflammatory skin disorder that primarily affects the central area of the face, including the cheeks, nose, chin and forehead. Its main clinical features are persistent erythema, papules, pustules, and telangiectasia. Rosacea affects approximately 5% of the adult population and it occurs both in men and women, with onset usually after the third decade of life [1]. The clinical manifestations of rosacea are highly variable, and its progression is typically irregular, with alternating periods of exacerbation and remission [2].

The National Rosacea Society (NRS) has classified rosacea into four main subtypes, which can overlap or progress from one form to another [3].

Erythematotelangiectatic rosacea (ETR) is the most common form, characterized by persistent redness (erythema) and visible blood vessels (telangiectasia) on the central face. Flushing is a common and recurrent symptom and often manifests as sudden episodes of redness and warmth in the facial skin. This subtype is most commonly linked to the early stages of rosacea, during which the inflammation is more widespread and less localized [4].

Papulopustular rosacea (PPR) is characterized by the appearance of papules and pustules, which may resemble acne vulgaris, with persistent central facial erythema and associated telangiectasias. Lesions involve periorificial regions such as the perioral, perinasal or periocular areas. Unlike acne vulgaris, it lacks comedones, which aids in differentiating between the two conditions [4]. 

Phymatous rosacea is characterized by skin thickening in specific areas of the face, caused by sebaceous gland hypertrophy and connective tissue proliferation [4]. While phymatous changes most commonly affect the nose, resulting in a condition known as ‘rhinophyma,’ they may also, though rarely, involve other areas such as the chin, forehead, cheeks, and ears. This condition is considered a later-stage manifestation of rosacea [4].

Ocular rosacea in characterized by the involvement of the eyes and it occurs in over 50% of rosacea patients. Symptoms include dryness, irritation, photophobia (sensitivity to light), conjunctivitis, and blepharitis (inflammation of the eyelids) [5]. In severe cases, ocular rosacea can lead to corneal inflammation and vision impairment. This subtype is often underdiagnosed because the ocular symptoms can be subtle or mistaken for other eye conditions [5].

These subtypes often overlap and the subtypes of rosacea can progress from one form to another: in its early stages, rosacea often presents with clinical presentation of ETR. As the disease progresses, patients may develop papules, pustules, telangiectasias, and more severe complications such as skin thickening and phyma [4].

**Figure 1:**
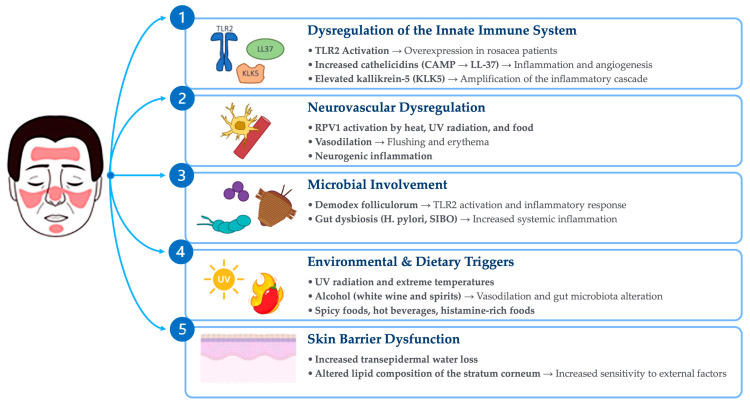
Main pathogenetic mechanisms of rosacea.

The roles of diet and gut microbiota in the pathogenesis of rosacea are important areas of research, with relevant implications for the therapeutic management of patients. Diet can affect rosacea through the direct effects of food metabolites and the modulation of the gut microbiota (Figure 1).

This narrative review critically evaluates the current evidence on the impact of diet and probiotics in rosacea, exploring their involvement in disease pathophysiology and in current therapeutic strategies. A total of 97 journal articles and reviews were analyzed, with a primary focus on studies published within the last twenty years, ensuring a focus on recent advancements in the field, while also considering some relevant earlier publications. The MEDLINE (PubMed), EMBASE and Cochrane library electronic databases were searched using the word “rosacea” plus one of the following keyword: “probiotics”, “gut-skin axis”, “microbiome” and “diet”. There was no limit to the search in terms of publication date, and the most recent search was run in December 2024. A manual search of reference lists was also performed. This article is based on existing research and does not present any new studies involving human participants or animals conducted by the authors.

### 1.2. Pathogenesis of Rosacea

Although recent advancements have provided substantial insights into the mechanisms underlying the disease, the pathogenesis of rosacea continues to be a subject of investigation. Multiple factors are thought to contribute to its development, including the dysregulation of the innate immune response and neuropeptide activity, microbial involvement, environmental factors, dietary triggers and skin barrier disfunction. Despite these advances, critical gaps remain in understanding the histopathological features of rosacea, particularly with regard to its subtypes and the age-dependent evolution of lesions [6,7]. A deeper understanding of these mechanisms is essential for developing targeted therapeutic strategies [8].

Innate immune system dysregulation plays a central role in the pathogenesis of rosacea, characterized by the overproduction of antimicrobial peptides like cathelicidins and pro-inflammatory cytokines. Cathelicidins are antimicrobial peptides expressed in the skin, notably overproduced in rosacea. In particular, human cathelicidin antimicrobial peptide (CAMP) plays a critical role in rosacea pathogenesis, exhibiting both immunomodulatory and angiogenic properties [8]. The production of cathelicidins is induced by the activation of TLR-2, receptors that are part of the innate immune system and are overexpressed on the skin of patients with rosacea. Patients with rosacea also exhibit abnormally high levels of kallikrein-5 (KLK5), a serine protease that processes CAMP into bioactive fragments such as LL-37, thereby amplifying the inflammatory cascade [9]. Elevated LL-37 levels stimulate the production of reactive oxygen species (ROS), pro-inflammatory cytokines (e.g., IL-8), and angiogenic factors (e.g., VEGF) via the epidermal growth factor receptor (EGFR) pathway [8,10]. These processes underlie the chronic inflammation and vascular dysfunction characteristic of rosacea [8,10].

Neurovascular dysregulation contributes significantly to rosacea symptoms. Activation of Transient Receptor Potential Vanilloid 1 (TRPV1) and related channels by environmental triggers like heat and UV radiation exacerbate flushing and erythema by promoting vasodilation and neurogenic inflammation. Enhanced sensitivity to these stimuli is a hallmark of the disease [11,12]. 

Within the skin, as in most organ systems, the microbiome is believed to play a role in facilitating proper immune function [13,14]. Emerging evidence suggests that while microorganisms may not be central causative factors in rosacea pathogenesis, alterations in the skin microbiome across multiple rosacea subtypes may act as trigger factors or potentiate inflammation in a subset of predisposed individuals, thereby contributing to disease progression [14]. One key aspect is the colonization of the skin by *Demodex folliculorum* mites, along with their associated bacteria, such as *Bacillus oleronius*. This microbial presence has the potential to activate Toll-like receptor 2 (TLR2) and consequently exacerbate the inflammatory processes characteristic of rosacea [14]. 

In addition to skin-related microbiota, gastrointestinal dysbiosis, including conditions such as *Helicobacter pylori* infection and small intestinal bacterial overgrowth (SIBO), has been hypothesized as a potential contributor to rosacea development. A higher prevalence of these conditions has been observed among rosacea patients, suggesting a possible causative connection between gastrointestinal dysbiosis and rosacea pathogenesis [15].

Environmental factors like temperature and UV radiation are common triggers for rosacea. Similarly, dietary factors, such as hot beverages, alcohol, spicy foods, and histamine-rich items, may exacerbate symptoms by stimulating TRPV1 channels and promoting vasodilation [16]. Avoiding these triggers may be beneficial and significantly reduce disease flares [17].

A significative association between rosacea and a compromised skin barrier function has been demonstrated. Increased transepidermal water loss and altered lipid composition in the stratum corneum contribute to dryness and sensitivity. These changes may amplify immune responses and increase susceptibility to environmental insults [18].

## 2. Gut Microbiota: Its Role and Implications in Dermatology

The gut microbiota may influence various dermatological diseases through a bidirectional interaction that involves immune, neuroendocrine, and metabolic pathways [19]. Both the skin and the gut are complex organs that are exposed to external environments. They have diverse microbiomes that are essential for maintaining homeostasis and organism survival [20,21]. The gut microbiome can have both beneficial and detrimental effects on the normal skin physiology [22,23]. The term “gut–skin axis” refers to the complex interactions between the gut and skin, primarily involving the regulation of systemic inflammation through complex mechanisms of immune system modulation [24,25]. 

Similar associations have been explored in many dermatological diseases, such as acne vulgaris, psoriasis, atopic dermatitis and hidradenitis suppurativa [20,26,27]. In the case of acne vulgaris, a disease that shares many clinical similarities with rosacea, recent studies have demonstrated reduced microbial diversity, lower levels of Firmicutes, and an increased abundance of Bacteroides [28]. Acne-prone skin, defined by its tendency for recurrent outbreaks of a few acne lesions along with subclinical alterations of the pilosebaceous units, shows susceptibility to clinical improvement following probiotic treatment [29]. In fact, we recently demonstrated that patients undergoing a maintenance therapy regimen with Lactobacillus probiotic supplementation showed a significant reduction in skin features that are involved in acne pathogenesis through a high-resolution non-invasive imaging technique [29].

Several gastrointestinal comorbidities, such as *H. pylori* infection, IBD, and small intestinal bacterial overgrowth (SIBO), have been linked to rosacea, suggesting that an altered gut microbiome may play an important role in its pathogenesis [15,30]. *H. pylori* exacerbates inflammation, triggering the release of pro-inflammatory cytokines such as TNF-α and IL-8. Additionally, it increases N_2_O production, promoting vasodilation and further amplifying the inflammatory response [31]. Few studies have highlighted specific alterations in the gut microbiota of rosacea patients, including compositional differences, reduced fecal microbial richness, and distinct microbial communities [32,33].

## 3. Diet and Rosacea

The role of diet in the pathophysiology and management of rosacea has gained increasing attention, with dietary triggers and beneficial nutrients recognized as important elements with high therapeutic potential (Table 1) [34]. Emerging research highlights the role of dietary factors in modulating the pathophysiology of rosacea through immune responses, neurovascular changes, and gut–skin interactions influencing the onset and progression of this dermatological condition [34,35]. Among the most well-documented dietary triggers, hot foods and beverages can exacerbate rosacea symptoms by inducing vasodilatation and stimulating transient receptor potential vanilloid member 1 (TRPV1) channels [36,37]. This mechanism contributes to flushing, heightened skin sensitivity and discomfort, reinforcing the need for dietary modifications in susceptible individuals [11,12]. Several foods are widely recognized as potential triggers for rosacea, among which alcohol is the most common [38], inducing vasodilation, inflammation, and oxidative stress. Alcohol metabolites such as acetaldehyde and acetic acid release histamine, exacerbating flushing and edema. Epidemiological evidence has linked alcohol intake—particularly white wine and liquor—with an increased risk of rosacea in women, with severity escalating in a dose-dependent manner [17,39]. Capsaicin, a compound found in spicy foods such as chili peppers, activates TRPV1 receptors in keratinocytes and nerve endings, leading to vasodilation and heat production [40]. However, a study by Yuan et al. found no causal link between spicy food consumption and the development of rosacea. Instead, spicy foods may exacerbate symptoms such as flushing, stinging, and burning without contributing to the condition’s onset [41]. In the same study, diets rich in dairy products were found to negatively correlate with rosacea severity, particularly in the ET and PPR phenotypes. Dairy may mitigate rosacea symptoms through its anti-inflammatory effects, potentially by modulating the gut microbiota and reducing intestinal inflammation [41]. Cinnamaldehyde, present in foods such as cinnamon, tomatoes, carrots, and chocolate, activates TRPA1 receptors, potentially aggravating rosacea symptoms. Similarly, histamine-rich foods, including fermented products, spinach, tomatoes, and certain fruits, can exacerbate symptoms by promoting vasodilation and inflammation, particularly in individuals with histamine intolerance, where diminished diamine oxidase activity leads to histamine accumulation and heightened sensitivity [18]. Niacin, or vitamin B3, found in foods such as liver, tuna, and peanuts, activates TRPV1 channels and niacin receptors, causing flushing and potentially worsening rosacea [42]. The relationship between caffeine and rosacea is complex. While caffeine’s vasoconstrictive, antioxidant, and immunosuppressive properties could theoretically mitigate rosacea symptoms, its potential role as a trigger remains debated [43,44]. Notably, a large cohort study reported an inverse association between coffee consumption and rosacea risk, suggesting a protective effect of coffee-derived caffeine [45].

High-fiber diets may support gut microbiota diversity and have anti-inflammatory effects, including on the skin [46]. Prebiotics, which selectively enhance beneficial gut bacteria, and probiotics, along with specific supplements, may help shift the gut microbiome toward a profile that supports skin health, potentially improving rosacea symptoms; however, the available evidence is not definitive, and more rigorous studies are needed [34,47]. Zinc, known for its anti-inflammatory and antioxidant properties, also supports skin health, with dietary sources including shellfish, dairy, and whole grains, although the effectiveness of zinc supplementation remains uncertain [48,49]. In a double-blind, placebo-controlled study, zinc sulfate 100 mg three times daily was found to be a good treatment for rosacea with significant improvements in the rosacea severity score [50]. Vitamin A, a fat-soluble micronutrient, influences various physiological and immunological processes with strong potential benefits on rosacea treatment. Robust studies and meta-analysis have investigated the use of topical and oral retinoids, especially isotretinoin, for rosacea treatment, with positive results [51,52].

**Table 1:** Relevant literature on rosacea and diet.

Author	Year	Country	Study Design	Rosacea Patient Number	Follow-Up	Investigation	Main Results and Conclusions
Alia E, Feng H 2022 [34]	2022	USA	Review	-	-	Dietary role in rosacea pathogenesis	Dietary factors influence rosacea pathogenesis and symptom management through TRPV channels, inflammation, and the gut–skin axis.
Weiss E, Katta R 2017 [35]	2017	USA	Review	-	-	Dietary role in rosacea management	This study highlights the potential of dietary interventions in managing rosacea by modulating inflammation and immune responses.
Li S, Cho E, Drucker AM, et al. 2017 [17]	2017	USA	Prospective cohort	4945 (total cohort: 82,737)	14 years	Alcohol intake and incidence of rosacea	Alcohol intake, especially white wine and liquor, is linked to increased rosacea risk in women.
Li S, Chen ML, Drucker AM, et al. 2018 [45]	2018	USA	Prospective cohort	4945 (total cohort: 82,737)	14 years	Caffeine intake and incidence of rosacea	There is an inverse association between coffee consumption and rosacea risk. This suggests a protective effect of caffeine.
Yuan X, Huang X, Wang B, et al. 2019 [41]	2019	China	Retrospective case–control	2637	2 years	Association between dietary factors and rosacea	Dairy product consumption is found to have a protective effect. Fatty foods and tea are significantly associated with an increased risk of rosacea. Sweet foods, spicy foods, and coffee show no significant association with rosacea.
Sharquie KE, Najim RA, et al. 2006 [50]	2006	Iraq	Double-blind, randomized, controlled trial	25	6 months	Efficacy and safety of oral zinc sulfate in treating rosacea	Zinc sulfate is potentially effective in rosacea management.

Overall, dietary strategies that avoid common triggers such as alcohol, spicy foods, and histamine-rich products, while emphasizing nutrient-dense options like zinc, omega-3 fatty acids, and fiber, may aid in symptom management. Further research is essential to develop robust dietary guidelines for rosacea patients [34].

## 4. Rosacea and Gastrointestinal Diseases

A growing body of evidence indicates a higher prevalence of gastrointestinal (GI) disorders, such as small intestinal bacterial overgrowth (SIBO), *Helicobacter pylori* (HP) infection, coeliac disease, Crohn’s disease and ulcerative colitis among patients with rosacea, suggesting a systemic inflammatory basis for the disease [53,54]. These findings suggest that GI diseases that compromise the integrity of the mucosal surfaces may contribute to the leaky gut phenomenon, exacerbate systemic inflammation and trigger rosacea symptoms [55]. One notable association was demonstrated between rosacea and small intestinal bacterial overgrowth (SIBO). Studies have found that patients with rosacea have a higher prevalence of SIBO compared to healthy controls, and the eradication of SIBO has been shown to improve rosacea symptoms [15]. This relationship underscores the role of microbial dysbiosis in the pathogenesis of rosacea, potentially mediated by systemic inflammation and gut barrier dysfunction [56]. 

The association between *Helicobacter pylori* (HP) infection and rosacea remains complex and influenced by several confounding factors [57]. The bacterium produces virulence factors, such as urease, that may increase systemic inflammation through the molecular mimicry and activation of pro-inflammatory cytokines [55]. A higher prevalence of *Helicobacter pylori* (HP) infection has been observed in patients with rosacea compared to the general population, with some patients reporting clinical improvement after eradication therapy [14]. However, the antibiotic treatment that is required for HP eradication represent an important confounding factor [58]. In fact, a recent meta-analysis found no significant effect of *HP* eradication on rosacea outcome [59], suggesting that the observed improvements may be attributed to the anti-inflammatory properties of antibiotics rather than HP eradication itself. This highlights the need for further investigation and clarification [60,61].

Rosacea has also been associated with other GI diseases, including coeliac disease, Crohn’s disease, and ulcerative colitis. A large nationwide cohort study of the Danish population found that patients with rosacea have a significantly higher risk of developing these conditions compared to the general population. Specifically, the study reported a 46% increased risk for celiac disease, a 45% increased risk for Crohn’s disease, and a 19% increased risk for ulcerative colitis [55]. The mechanism linking rosacea and inflammatory bowel disease (IBD) remains unclear; however, both are chronic inflammatory diseases associated with the dysregulation of innate and adaptive immunity, driven by a complex interplay of genetic and immunological factors [62]. These findings should prompt clinicians to consider a comorbid IBD diagnosis in rosacea patients who present with chronic abdominal pain, prolonged diarrhea, or bloody stools [63].

The bidirectional nature of the gut–skin axis suggests that improving gut health may provide therapeutic benefits for rosacea. Further studies are needed to elucidate the exact mechanisms connecting GI microbiota to rosacea and to develop effective gastrointestinal interventions that benefit rosacea patients [64].

## 5. Probiotics in Rosacea

Despite the availability of various therapies, managing rosacea remains challenging due to frequent relapses and persistent symptoms [65]. Emerging evidence suggests that probiotics, defined as live microorganisms that confer health benefits when administered in adequate amounts, may play a role in managing rosacea through their influence on the gut–skin axis as an effective adjunctive treatment with few side effects (Table 2) [65,66].

Their therapeutic use is justified by their action on gut dysbiosis and by promoting a healthier microbial balance, which may help reduce systemic inflammation and enhance skin conditions [67,68].

This therapeutic strategy leverages the complex interplay between gut and skin health, targeting systemic inflammation and barrier dysfunction that are commonly associated with a variety skin disorders, particularly rosacea [67,69].

Since probiotics act on the gut microbiota, their mechanism of action includes the following: modulating the immune response, reducing neurogenic inflammation, improving skin barrier function, enhancing barrier restoration after damage; and reducing vasodilation, edema, mast cell breakdown and TNF-a release [70]. For example, some strains of *Lactobacilli* have been shown to inhibit substance P-induced skin inflammation and improve skin barrier recovery, reducing skin sensitivity. In addition, probiotics reduce transepidermal water loss (TEWL) and improve skin hydration, both of which are impaired in patients with rosacea [67,69]. Several studies have explored the potential of probiotics in managing rosacea. Evidence has shown that the oral use of probiotics, such as *Escherichia coli Nissle*, a mixture of *Bifidobacterium* strains and a mixture of *Bifidobacterium* and *Lactobacillus* in combination with standard topical and systemic therapies, leads to more frequent clinical remission, substantial symptom improvement, and a reduction in relapses lasting up to six months after the initiation of therapy [68,71,72]. *Bifidobacterium* strains and *S. boulardii* attenuate inflammatory factor release by increasing interleukin (IL)-10 production and suppressing NF-κB, inducible nitric oxide synthase (iNOS), p65, and IL-17 A [73]. Recent studies indicate that *S. boulardii,* both probiotic and postbiotic (heat-killed), are able to mitigate or even restore alterations of the composition, structure, and functionality of the intestinal microbiota to normal levels in experimental inflammatory gut conditions [74,75].

Recently, topical probiotics have been studied as an alternative to oral formulations for the treatment of skin conditions [76]. Interestingly, a formulation containing *Vitreoscilla filiformis* probiotic fractions demonstrated notable reductions in erythema, Demodex density, and transepidermal water loss, as well as improvements in patient-reported outcomes. These findings indicate that topical probiotics may directly enhance skin barrier function and modulate immune responses locally [77]. 

Despite promising results, the evidence supporting the use of probiotics in the treatment of rosacea remains relatively limited. Further clinical trials are necessary to evaluate and compare the effectiveness of various probiotic strains and different methods of delivery, such as oral ingestion and topical use. Furthermore, it has been observed that the beneficial effects of probiotics tend to decrease once their use is discontinued, highlighting the importance of ongoing or repeated treatments to maintain their positive impact [70,78].

**Table 2:** Relevant literature on rosacea and probiotics.

Author	Year	Country	Study Design	Patient Number	Follow-Up	Investigation	Results and Conclusions
Gueniche et al. 2010 [67]	2010	France	In vitro study	-	-	*Lactobacillus paracasei* CNCM I-2116 (ST11)	*Lactobacillus paracasei* CNCM I-2116 (ST11) inhibit skin inflammation induced by substance P and recover skin barrier function.
Buianova et al. 2018 [68]	2018	USA	Review article	-	-	Role of intestinal microbiome and probiotics in pathophysiology of rosacea	Probiotics may be a potential treatment option for rosacea.
Manzhalii et al. 2016 [71]	2016	Germany	Clinical trial	82	1 month	*Escherichia coli Nissle 1917* on several dermatoses, including rosacea	*Escherichia coli Nissle 1917* improves several skin diseases.
Fortuna et al. 2016 [72]	2016	Italy	Case report	1	6 months	Effect of combining low-dose doxycycline and probiotic therapy in treating scalp rosacea	The combination of doxycycline and probiotics improves symptoms in a patient with rosacea of the scalp.
Knackstedt et al. 2020 [77]	2020	NS	Review article	-	-	Use of topical probiotics in treating skin conditions, including rosacea	Topical probiotics have anti-inflammatory and skin barrier-repairing properties.
Pinchuk IV, Bressollier P, Verneuil B, et al. 200 [78]	2001	France	In vitro study	-	-	Role of *Bacillus subtilis 3* on *Helicobacter pylori* and its potential impact on rosacea	*Bacillus subtilis 3* shows significant anti-*H. pylori* activity.

## 6. Conclusions

Studies and reports over the past several decades have analyzed the association between common dietary triggers and rosacea pathogenesis. The avoidance of common triggers can reduce rosacea flares and improve the management of the disease. Probiotics, such as certain strains of *Lactobacillus*, *Bifidobacteria* and *Saccharomyces,* can positively influence several immune mechanisms that are implicated in rosacea pathogenesis by increasing IL10 and reducing TNF-a, IL-17A. Prebiotics, which supports gut colonization by beneficial gut bacteria, could favorably influence the gut–skin axis mechanisms involved in rosacea pathogenesis. To the best of our knowledge, the paucity of clinical studies and the lack of randomized trials and standardization of the use of probiotics are major limitations to the current adoption of probiotics as part of standard rosacea care and management. Diet and nutritional counseling may enhance rosacea management through the direct effects of food metabolites and the modulation of the gut microbiota. High-fiber diets, particularly those rich in vitamin A, may exhibit potent anti-inflammatory and sebum-regulating properties that help to manage the factors contributing to the progression of rosacea. Therefore, modulating the skin and gut microbiota through diet, prebiotics, probiotics, and postbiotics could represent an effective and innovative strategy for the therapeutic control and management of rosacea. However, few studies are available, and more rigorous clinical trials are needed.
